# Tuning Polyolefin Hydrophilicity to Control Sulfonation Cross-Linking Kinetics for Carbon Synthesis

**DOI:** 10.1021/acsaenm.5c00210

**Published:** 2025-05-15

**Authors:** Carmen B. Dunn, Zoe Gunter, Anthony Griffin, Paul Smith, Ahmed Al-Ostaz, Mine G. Ucak-Astarlioglu, Zhe Qiang

**Affiliations:** † School of Polymer Science and Engineering, University of Southern Mississippi, 118 College Drive, Hattiesburg, Mississippi 39406, United States; ‡ Department of Civil Engineering, 8083 University of Mississippi, Mississippi 38677, United States; § US Army Engineer Research and Development Center, 3909 Halls Ferry Road, Vicksburg, Mississippi 39180-6199, United States

**Keywords:** polyolefin, tunable hydrophilicity, functionalized carbon precursors, sulfonation-induced crosslinking, reaction kinetics, carbon conversion

## Abstract

The use of polyolefins as carbon precursors has gained significant attention due to their low cost, wide availability, and potential for upcycling waste materials. A critical step in this process is using sulfuric acid as the chemical agent for sulfonation-enabled crosslinking of polyolefins, which forms thermally stable networks to allow their effective conversion into carbon upon pyrolysis. However, the hydrophobic nature of most commodity semicrystalline polyolefins often limits acid diffusion, making the reaction sluggish. This work investigates the impact of improving polyolefin hydrophilicity to enhance its sulfonation kinetics. By grafting hydroxyl groups onto the polyolefin backbone, we achieved significant enhancement in reaction efficiency. Specifically, just 2 h of crosslinking enables hydroxyl-functionalized, hydrogenated polybutadiene (hPB) to achieve over 50 wt% carbon yield after carbonization, whereas hPB requires 16 h of crosslinking to reach a comparable yield. These results highlight the significance of polyolefin carbon precursor design in overcoming diffusion limits, accelerating sulfonation kinetics, and improving carbon conversion efficiency, further advancing their use for carbon material production.

## Graphical abstract

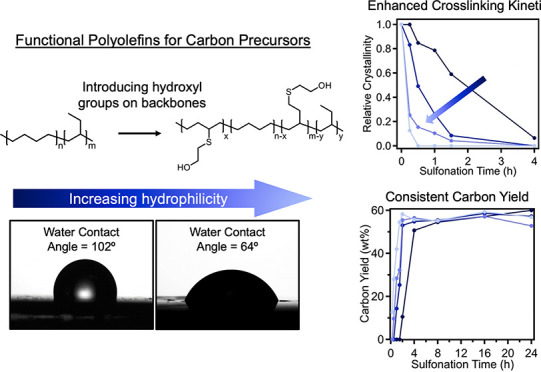

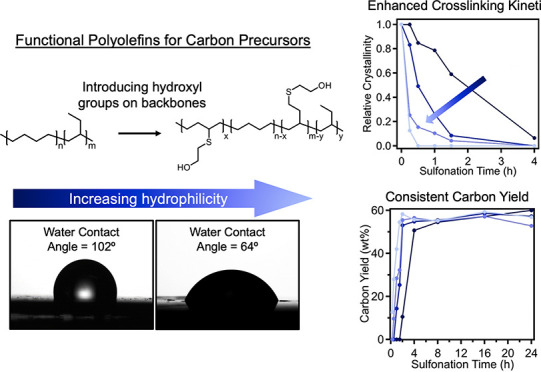

## Introduction

The development of porous carbon materials has been extensively studied over the past few decades due to their broad range of applications, including energy storage,^1–4^ water purification,^5–8^ and catalyst support.^9–12^ While various biomass materials have been broadly utilized as precursors to produce biochar with high surface areas through direct pyrolysis, they often present a challenge of inconsistent feedstock quality across different regions, leading to variability in the final material properties.^13,14^ Additionally, direct pyrolysis of biomass-based precursors may pose difficulties in achieving uniform porosity and controlled performance metrics. Alternatively, several synthetic polymers have been established as efficient carbon precursors, including polyacrylonitrile (PAN),^15–19^ pitch,^20,21^ phenolic resin,^22–24^ polyolefins,^25–28^ and others.^29–31^ In general, the formation of thermally stable networks from these precursors is essential to enable their efficient conversion into carbon. Additionally, pyrolysis of crosslinked polymer precursors often leads to pore formation in the carbon framework due to factors such as kinetically limited densification during pyrolysis, density differences between the precursor and the resulting carbon structure, as well as variations in carbon yield. In many systems, the inherent porosity from pyrolysis of polymer precursors allows for the tailored design of porous carbon materials for specific applications, providing advantages in consistency and scalability over those of their biomass-derived alternatives.

Pioneering work by Horikiri et al. and Postema et al., respectively, demonstrated the ability to produce carbon fibers from polyolefins, which relies on a necessary step of transforming linear polyolefin chains into crosslinked networks.^32,33^ Among the various crosslinking methods, the most widely used approach involves directly immersing polyolefins in concentrated sulfuric acid at elevated temperatures, typically exceeding 100 °C, to induce aromatization of their polymer backbones through sulfonation. Specifically, the mechanism underlying sulfonation-induced crosslinking in polyethylene (PE) has been systematically studied through a combination of experimental and computational approaches, revealing that the reaction involves both five-centered internal elimination and radical chain reactions, which generate the crosslinking sites.^34,35^ It is important to note that during this process, sulfuric acid needs to penetrate throughout the precursor material via a diffusion mechanism, while the macroscopic morphology of the polyolefin precursors can be retained under optimal reaction conditions.

Significant efforts have been dedicated to advancing polyolefins as a cost-effective solution for synthesizing carbon, which also presents a promising approach for waste management. For example, sulfonation-based crosslinking allows the upcycling of polyolefin textiles to carbon fibers,^36^ which show great potential for energy storage and environmental remediation, including serving as micropollutant sorbents, electrodes for sodium-ion batteries, and resistive heaters for energy-efficient processing.^36–41^ We note that this research area continues to attract interest due to the potential of low-cost, widely available polyolefins and their waste to enable functional carbon production through scalable and economically viable processes. However, a fundamental limitation of crosslinking polyolefin-based precursors using sulfuric acid is their diffusion-controlled nature, which often leads to sluggish reaction kinetics due to the hydrophobicity of most commodity polyolefins. As a result, scaling up these reactions to large-scale systems remains a key challenge, as it requires extended reaction times. Interestingly, recent studies have revealed that the sulfonation crosslinking of thick polyolefin fibers could involve a cracking mechanism that can facilitate the diffusion of acid through microcrack channels.^40,41^ Specifically, sulfonation alters the hydrophilicity and swelling behavior of polyolefins, while unreacted regions remain hydrophobic and intact. This mismatch in volumetric expansion generates internal stresses, leading to microcrack formation. Although this mechanism accelerates reaction times for large-scale polyolefin components from hundreds of hours to tens, it inherently causes crack formation, which can be undesirable for many applications. To fully unlock the potential of polyolefins for carbon production, it is crucial to develop simple and effective methods that ensure efficient reaction kinetics while limiting crack formation in the final material.

In this study, we demonstrate the synthesis of hydroxyl-functionalized polyolefins as carbon precursors, designed to enhance hydrophilicity and improve the diffusion of sulfuric acid during the sulfonation cross-linking reaction. By increasing hydroxyl functional group content on semicrystalline polyolefin backbones, we observe accelerated reaction kinetics, collectively driven by enhanced hydrophilicity and reduced crystallinity, while preserving relatively high carbon yields comparable to those reported in prior studies.^36,41^ Our study also explores the impact of hydroxyl group content on the porosity and morphology of the resulting carbon materials. These findings provide key insights into the rational design of polyolefins for optimal carbon synthesis, paving the way for the more efficient and scalable production of high-performance carbon materials.

## 2 Experimental Section

### 2.1 Materials

1,3-butadiene (20 wt % in toluene) and *sec*-butyl-Li (1.4 M in cyclohexane) were purchased from Sigma. Tetrahydrofuran (THF) (HPLC grade), methanol (ACS grade), and xylenes (ACS grade) were obtained through Fisher Chemical. Mercaptoethanol and butyl hydroxytoluene were purchased from TCI. Azobis­(isobutyronitrile) (AIBN), p-toluenesulfonyl- hydrazide, tributylamine, and sulfuric acid (95–98%) were all acquired from Sigma-Aldrich. Deionized (DI) water was obtained from a Millipore Milli-Q filtration system.

### 2.2 Synthesis of Hydrogenated Functional 1,4-Polybutadiene (hPB)

Under N_2_ atmosphere in a glovebox, 20 wt % 1,3-butadiene in toluene (37.2 mL, 1 equiv) was measured and added to a 300 mL high-pressure reactor equipped with a magnetic stir bar. Subsequently, 1.4 M *sec*-butyl-lithium (0.25 mL, 0.0032 equiv) was introduced to the reactor. The reactor was immediately sealed, removed from the glovebox, and placed in an oil bath of 40 °C stirring at 200 rpm for 5 h. Upon completion, the reactor was opened, and the mixture was quenched with excess amount of methanol. The contents of the flask were then concentrated to half volume before precipitation into cold methanol. A clear, colorless, viscous liquid product was obtained which was dried under vacuum for 20 h.

The 1,4-polybutadiene was functionalized via a thiol–ene coupling reaction. Specifically, a three-neck round-bottom flask equipped with a magnetic stir bar was charged with 1,4-polybutadiene (3 g, 1 equiv) and 35 mL of THF. This mixture was purged with N_2_ for 10 min before the addition of mercaptoethanol (7.8 mL, 2 equiv). Upon N_2_ purge for an additional 5 min, AIBN (45.6 mg, 0.005 equiv) was added to the reaction vessel, which was submerged in a 60 °C oil bath with stirring. At time increments of 3, 12, and 30 min, a third of the initial reaction volume was quenched by directly pouring into methanol containing excess amount of butylated hydroxytoluene. The methanol from the precipitation of the functionalized 1,4-polybutadiene was decanted off, and a reaction aliquot of functionalized polymer (3 g, 1 equiv) was dried under vacuum for 20 h. Each aliquot was then transferred to a 250 mL round-bottom flask equipped with a Teflon magnetic stir bar.

For the hydrogenation of polybutadiene, the polymer was dissolved in 100 mL of xylenes, and tributylamine (39 mL, 3 equiv), p-toluenesulfonyl hydrazide (28.9 g, 2.8 equiv), and butyl hydroxytoluene (40 mg, 0.0025 equiv) were then added to the reaction flask, which was equipped with a condenser and submerged in an oil bath for 20 h at 140 °C. Upon completion of this reaction, the contents of the round-bottom flask were precipitated into excess of methanol and gravity filtered. The product was reprecipitated and filtered twice before drying under a vacuum for 20 h to obtain a white solid product. Note, we refer to hydrogenated 1,4-polybutadiene as hPB and three samples of increasing hydroxyl content as hPB-*g*-OH1, hPB-*g*-OH2, and hPB-*g-*OH3.

### 2.3 Sulfonation-Induced Cross-Linking and Carbonization of Functionalized Polyolefin

All hPB and hPB-*g-*OH samples were compression-molded into films with an average thickness of 0.47 mm at 110 °C. They were then immersed in concentrated sulfuric acid (95–98%) and transferred to a Thermo Scientific Thermolyne FB1315 M muffle furnace at 135 °C. After reacting for times ranging from 15 min to 24 h, samples were removed, filtered, and washed with DI water to remove residual acid. After drying in a vacuum oven at 120 °C for 24 h, samples were either characterized or transferred to an MTI GSL-1100× tube furnace for carbonization under N_2_ atmosphere, where a ramp rate of 1 °C/min to 600 and 5 °C/min to 800 °C was used. After carbonization, samples were allowed to cool to ambient temperatures before being removed and characterized.

### 2.4 Characterizations

1,4-polybutadiene was characterized via gel permeation chromatography (GPC) to determine the number-average molecular weight (*M*_n_), the weight-average molecular weight (*M*_w_), and the polydispersity index (*Đ*) relative to the polystyrene standards at 45 °C in THF, using a Waters ACQUITY Advanced Polymer Chromatography system equipped with ACQUITY APC XT 450, ACQUITY APC XT 1225, and APC XT 45 columns in series. 1,4-Polybutadiene was also characterized by ^1^H Nuclear Magnetic Resonance (NMR) spectroscopy using a Bruker Ascend 400 spectrometer (Bruker Corporation, Billerica, Massachusetts, USA) at a 400 MHz proton frequency and a temperature of 303 K. All samples were dissolved in deuterated chloroform (CDCl_3_) at a concentration of 40 mg/mL. The ^1^H NMR experiments were performed with 16 scans and a proton relaxation decay of 3 s. All data was processed using Topspin 3.7 software.

Attenuated Total Reflectance Fourier Transform Infrared (ATR-FTIR) spectroscopy of the hPB and hPB-*g*-OH samples before and after sulfonation-induced cross-linking was performed on a Thermo Scientific Nicolet 6700 FT-IR spectrometer with a SMART iTR stage attachment at a resolution of 2.0 cm^–1^ with 32 scans and a scanning wavenumber in the range of 4000–600 cm^–1^. A background of 16 scans with the same resolution was collected prior to each measurement. For water contact angle measurements, hPB samples were dissolved in xylenes to form 5 wt % solutions which were then drop cast onto silicon wafers. The resulting films were vacuum-dried to ensure the removal of all solvent. Water contact angle (WCA) was measured using 4–6 μL water droplets with a Ramé-Hart 200–00 Std. goniometer. An ImageJ Drop Analysis was used to analyze the droplets and obtain static contact angle measurements. All materials were analyzed with the water contact angle measured in triplicate.

Differential scanning calorimetry (DSC) was conducted to determine the degree of crystallinity (*X*_c_) and relative *X*_c_ of the pristine and cross-linked polyolefins, respectively, using a TA Instruments Discovery 250 DSC. For the pristine polyolefins, heat–cool-heat measurements were conducted under nitrogen at a ramp rate of 10 °C/min from −90 to 250 °C. For the cross-linked samples, a heat–cool-heat cycle was performed under nitrogen ramping 10 °C/min from ambient to 250 °C, 5 °C/min to 20 °C, and 10 °C/min to 250 °C. The second heating scan was used to determine *X*_c_ with a reference enthalpy of 293 J/g.^42^ Data analysis was conducted on TRIOS.

Moreover, carbon yield calculations of these samples were determined by comparing the mass of the carbonized samples to the initial mass of their precursor samples before sulfonation. A Micromeritics Tristar II 3020 was employed to perform nitrogen physisorption measurements at 77 K with relative pressures ranging from 0.0000512 to 1 (*p*_0_ = 1 atm). Samples were degassed under a vacuum at a temperature of 325 °C overnight before sample analysis was performed. Pore size distribution analysis was executed using nonlocal density functional theory (NLDFT) analysis (within the Micromeritics TriStar II 3020 software) with a carbon slit model, as it is commonly used to characterize amorphous carbon materials, and surface area and pore volume calculations were executed using Brunauer–Emmett–Teller (BET) analysis and the t-plot method with silica–alumina as the standard, respectively. A Zeiss Ultra 60 field-emission scanning electron microscope (SEM) was utilized to record high-resolution micrographs of the functionalized and cross-linked polyolefin material surfaces, employing an accelerating voltage ranging from 3.0 to 20.0 kV.

## 3 Results and Discussion

As shown in Figure 1, the synthesis of semicrystalline polyolefins with controlled hydrophilicity was achieved through a batch process, beginning with 1,4-polybutadiene (1,4-PB) as the base material, followed by thiol–ene coupling and subsequent hydrogenation to produce semicrystalline polymers. These steps are straightforward, making this approach potentially promising for the scaled production of functionalized polyolefins as carbon precursors. In the thiol–ene reaction, a small amount of AIBN initiator generates free radicals, while the reaction time can be varied to control the amount of hydroxyl group installation on the polyolefin backbone, directly influencing its hydrophilicity. Specifically, we used a reaction time of 3, 12, 30 min for preparing PB*-g*-OH1, PB*-g*-OH2, and PB*-g*-OH3, respectively, allowing an increased content of hydroxyl grafts on polymer backbones with longer reaction times. The successful synthesis of 1,4-PB as the starting material was confirmed by ^1^H NMR spectroscopy. As shown in Figure 2a, the peak at 5.0 ppm corresponds to the alkene region of butadiene monomers added through vinyl addition (1,2-microstructure), and the peak at 5.5 ppm is associated with the alkenes within the backbone (1,4-microstructure). Through integrating these regions, the ratio of 1,2/1,4 monomer addition can be determined, which contains approximately 89 mol % 1,4-addition (Figure 2a) and is consistent with a previous report.^43^ Moreover, GPC was employed to study the molecular weight of the polybutadiene precursors; their *M*_n_ was approximately 17 ,000 g/mol with a narrow *Đ* of 1.03 (Figure 2b).

**Figure 1:**
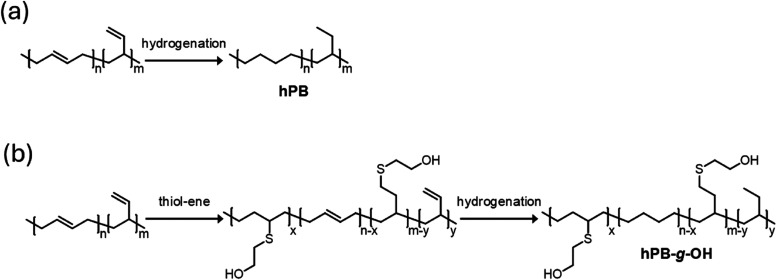
(a) Synthetic route to obtain hPB through hydrogenation of PB. (b) Synthetic route to obtain hPB-*g-*OH through thiol–ene coupling and hydrogenation steps.

**Figure 2:**
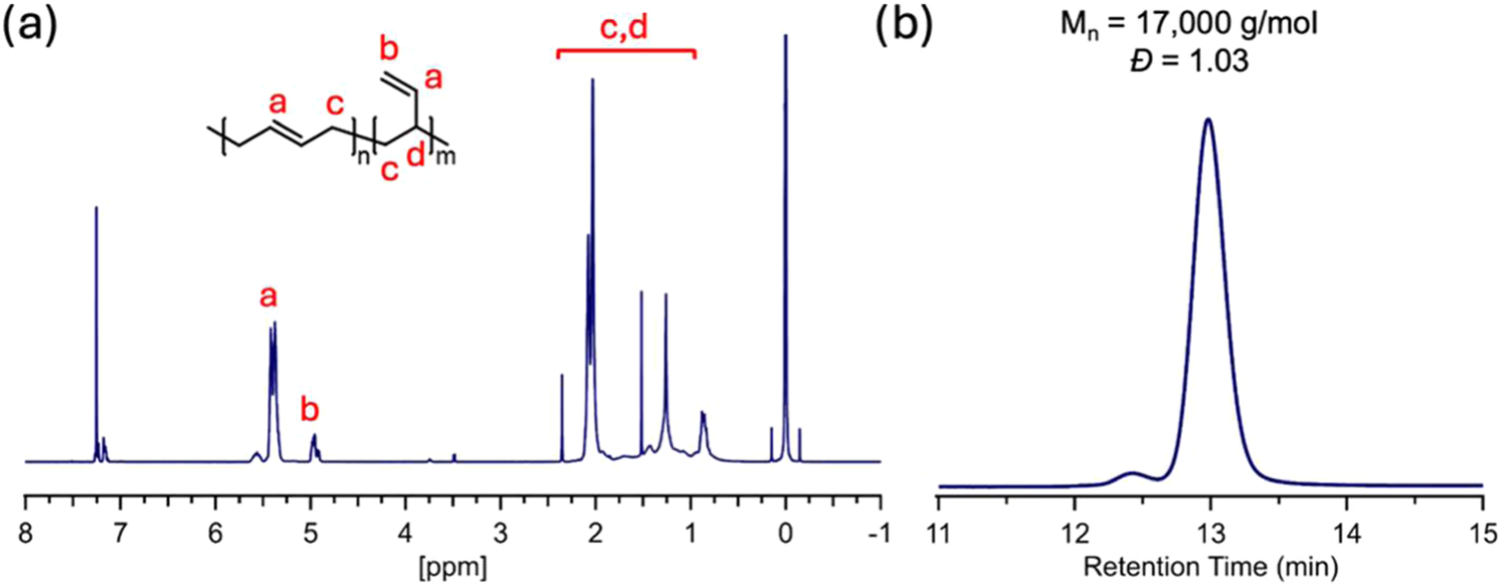
(a) ^1^H NMR spectra of polybutadiene showing predominant 1,4-addition microstructure, (b) their corresponding GPC trace.

The chemical composition of hPB and hPB-*g-*OH materials was investigated using FTIR spectroscopy. As shown in Figure 3a, the increasing intensity of a broad band in the FTIR spectrum at 3200 cm^–1^, corresponding to hydroxyl groups, indicates an enhanced grafting density from sample hPB to hPB-*g-*OH3. Additionally, the near disappearance of alkene stretching vibrations at 1350 cm^–1^, coupled with the gradual emergence of the C–O asymmetric stretching vibration at 1235 cm^–1^, confirms the successful grafting and hydrogenation of hydroxyl functionalities in varying amounts. These spectral changes confirm the efficient functionalization of the 1,4-polybutadiene backbone with hydroxyl groups at a controlled graft density. During the thiol–ene grafting of mercaptoethanol, extending the reaction time allows for an increased amount of hydroxyl groups onto the polymer backbone, demonstrating a direct correlation between reaction time and functionalization extent. We further studied the impact of hydroxyl group content on the hydrophilicity of these polyolefin materials using WCA measurements. Figure 3b shows that the hPB exhibits a WCA of ∼102.1°, which is consistent with previously reported literature results.^44^ Upon introduction of hydroxyl groups on their backbones at varied content, it is found that their WCA can decrease to ∼94.9, ∼72.5, and ∼63.6° for hPB-*g-*OH1, hPB-*g*-OH2, and hPB-*g-*OH3 samples, respectively. These results show that longer reaction times for thiol–ene grafting lead to the formation of more hydrophilic polyolefins. It is worth noting that the 17, 000 g/mol PB is of sufficient molecular weight to undergo intermolecular cross-linking upon sample drying and during storage, due to the presence of unsaturated bonds in the polymer backbones; thus, these PB–OH materials were immediately hydrogenated. In Figure S1, we showed the ^1^H NMR spectra of PB–OH samples derived from another parent polymer of 1,4-PB (32 ,000 g/mol), as well as their corresponding water contact angle results. Based on these findings, we anticipate that the hydroxyl functionalization produced PB*-g*-OH between 5 and 10 mol %. Our method provides a simple approach to synthesize semicrystalline hPB with tunable hydrophilicity, enabling its tailored use as a carbon precursor.

**Figure 3:**
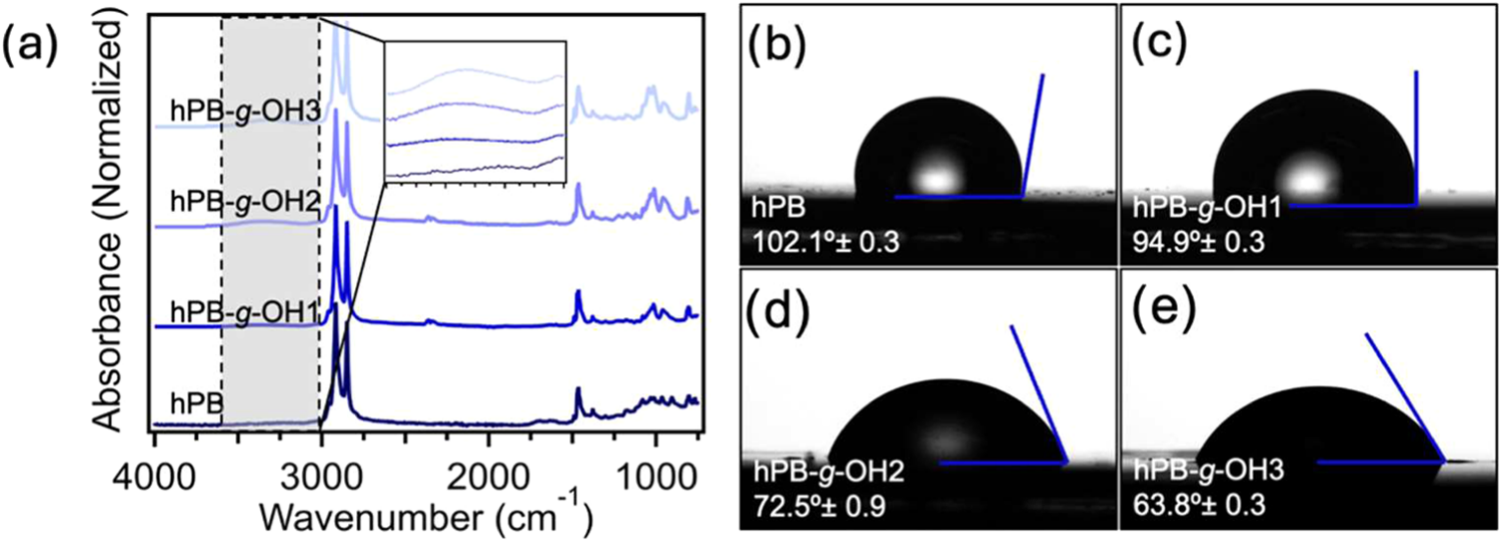
(a) FTIR spectra of hPB, hPB-*g-*OH1, hPB-*g-*OH2, and hPB-*g-*OH3 normalized by alkane peak at 2848 cm^–1^ indicating an increase in −OH stretching and C–O stretching vibrations upon extending thiol–ene reaction time. WCA measurements for (b) hPB, (c) hPB-*g-*OH1, (d) hPB-*g-*OH2, and (e) hPB-*g-*OH3 with decreasing WCA.

Furthermore, the impact of the hydroxyl group content on the crystalline behaviors of hPB, hPB-*g*-OH1, hPB-*g-*OH2, and hPB-*g*-OH3 was investigated using DSC experiments (Figure 4a). For neat hPB, a melting temperature of 90.5 °C and a melting enthalpy of 67.8 J/g was found. These values are lower than typical polyolefin materials such as low-density polyethylene (PE). We attribute this difference to the microstructure of our hPB. We note that while the 1,4-addition can contribute to the semicrystalline nature of the polyolefin, the 1,2-addition units, which make up approximately 11 mol % of the hPB composition, can disrupt crystallization. Since the crystallizable unit of hPB and hPB-*g-*OH materials should be identical to that of PE, a reference enthalpy value of 293 J/g was used to calculate the degree of crystallinity (*X*_c_) for each material. Consequently, the degree of crystallinity of hPB is reduced to about 20%. For hPB-*g*-OH samples, the hydroxyl group can further influence crystallinity and melting point. Specifically, hPB-*g-*OH1, hPB-*g*-OH2, and hPB-*g-*OH3 have melting temperatures of 87, 82, and 68 °C, respectively (Figure 4b). The DSC thermogram of these materials, illustrating their crystallization behavior, is presented in Figure S2. The decrease in melting temperature upon the introduction of additional functional groups is consistent with previous findings.^45,46^ For example, Li et al., demonstrated that functionalization of polyolefins with amides creates sites for hydrogen bonding, which disrupts the regular polymer chain packing and, consequently, reduces the melting temperature and crystallinity.^45^ Additionally, the melt enthalpies for the hPB-*g*-OH samples were measured at 37.9, 31.8, and 17.6 J/g with increasing hydroxyl content. These findings indicate that the addition of hydroxyl functional groups on the polymer backbone inhibits chain packing, reducing the formation of crystalline structures, as evidenced by the decreasing degree of crystallinity. Furthermore, the observed decrease in the melting temperature with higher hydroxyl grafting suggests the formation of smaller size crystal domains. These results highlight the significant impact of hydroxyl functionalization on the crystalline structure and thermal properties of these hPB materials.

**Figure 4:**
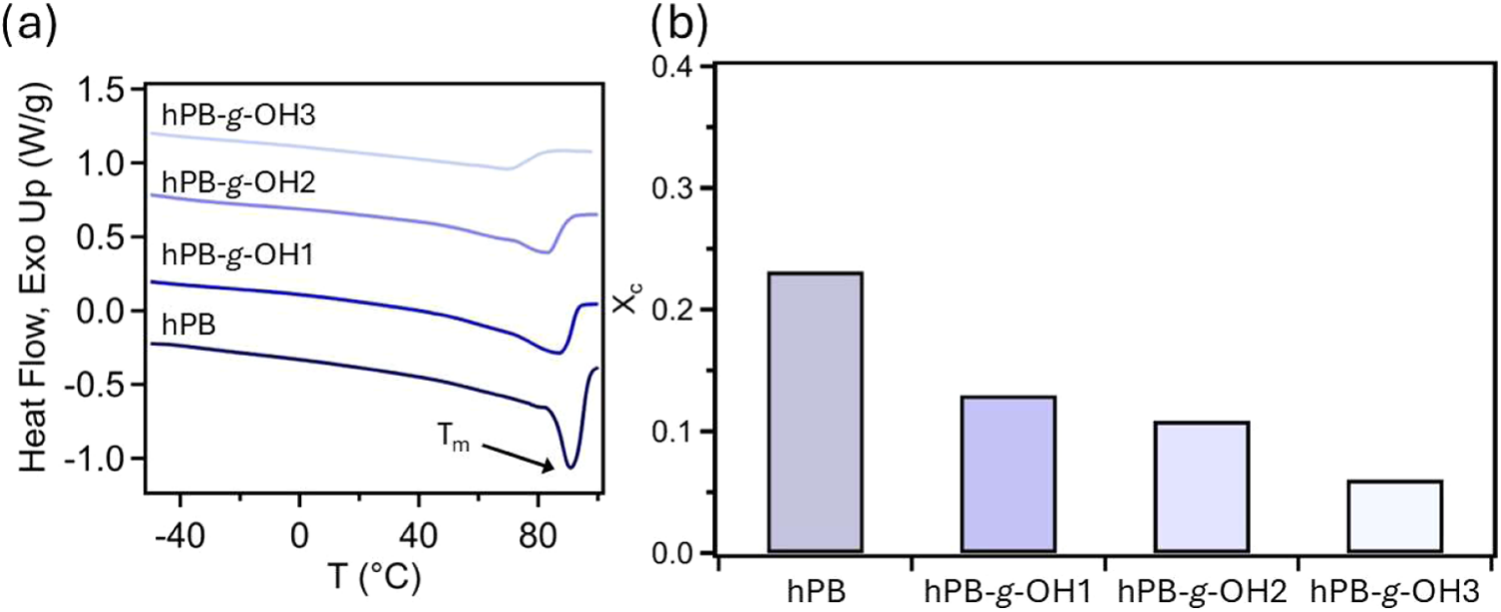
(a) From the bottom, melting curves of hPB, hPB*-g*-OH1 (offset by 0.5 W/g), hPB-*g-*OH2 (offset by 1 W/g), and hPB-*g*-OH3 (offset by 1.5 W/g) from DSC heat–cool-heat experiments (second heating cycle) and (b) measured degrees of crystallinity for hPB, hPB*-g*-OH1, hPB*-g*-OH2, and hPB-*g-*OH3 indicating a decrease in crystallinity with the addition of more hydroxyl grafts.

A simplified reaction scheme illustrating the sulfonation-induced crosslinking reaction of hPB and hPB-*g*-OH materials is presented in Figure 5, which is a key step required to enable their use as carbon precursors, preventing excessive thermal degradation and preserving overall material structural integrity. This crosslinking process first involves the chemical attachment of sulfonic acid groups onto the polymer backbone, followed by olefination, resulting in the formation of unsaturated bonds. As the material continues to react with sulfuric acid, radical species can be generated which undergo a coupling reaction to form intermolecular cross-linked structures. As most commodity polyolefins are hydrophobic, the crosslinking step is often diffusion-controlled, requiring acid penetration to fully react with the polyolefin precursor.^47,48^ Specifically, as the reaction progresses, portions of the sample become hydrophilic, facilitating acid diffusion. However, this process can be slower in semicrystalline precursors compared with their amorphous counterparts, further hindering reaction efficiency.

**Figure 5:**
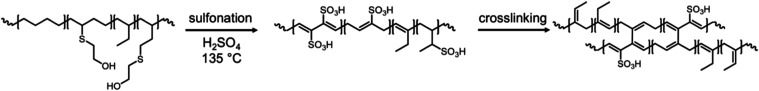
A simplified reaction scheme demonstrating sulfonation-induced cross-linking of hPB-*g-*OH materials.

Figure 6 shows the change in relative degree of crystallinity (*X*_c_) for each sample as a function of sulfonation-induced cross-linking reaction time at 135 °C, which can be used to assess their reaction kinetics. The raw DSC thermographs of these samples are included in Figure S3. Specifically, the loss of crystallinity in these samples with reaction progression can be attributed to both the formation of intermolecular cross-links and the addition of bulky sulfonic groups, which restrict the ability of the hPB and hPB-*g*-OH materials to recrystallize. For hPB, the degree of crystallinity decreased at a relatively slower rate with increasing sulfonation time, becoming nearly amorphous after at least 4 h of sulfonation. In contrast, the hPB*-g*-OH samples exhibited a significantly faster reduction in crystallinity. The relative crystallinity of hPB-*g*-OH1 approached 0% after 4 h, hPB-*g*-OH2 after 1.5 h, and hPB-*g*-OH3 after just 30 min of sulfonation. Previous studies suggest that introducing macropores in polyolefin precursors can enhance acid diffusion, thereby improving their cross-linking kinetics.^49^ However, in this study, all hPB and hPB-*g*-OH precursors were compression-molded into solid films with a thickness of 0.47 mm. Therefore, the observed changes in cross-linking kinetics are primarily attributed to acid diffusion within the bulk. It is noteworthy that the reaction temperature of 135 °C is above the melting temperature of these samples. These results demonstrate that increasing the hydroxyl content enhances the hydrophilicity of the hPB-*g*-OH materials, allowing for improved diffusion of sulfuric acid into the polymer matrix. This enhanced acid penetration significantly accelerates the sulfonation-induced cross-linking reaction, leading to faster kinetics. To further evaluate the chemical structure of the samples after sulfonation and confirm the formation of cross-links, FTIR spectra of samples sulfonated for 24 h were recorded and analyzed prior to carbonization (Figure S4). Specifically, the reduction of alkyl stretching bands (∼2880 and ∼2940 cm^–1^) and the appearance of S = O and C = C stretching bands at ∼1170 and ∼1600 cm^–1^ are observed, which indicate the addition of sulfonic acid groups and the formation of unsaturated bonds, respectively.

**Figure 6:**
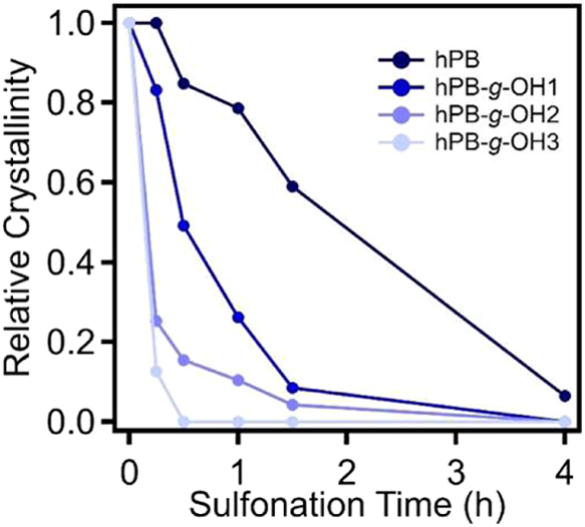
Relative *X*_c_ of hPB, hPB-*g-*OH1, hPB-*g-*OH2, and hPB-*g*-OH3 (compared to their initial state) as a function of the sulfonation time at 135 °C. All samples were in the film state with an average thickness of 0.47 mm.

Moreover, SEM was employed to characterize the surface morphology of hPB and hPB-*g*-OH samples before (Figure S5) and after sulfonation (Figure 7). Each material was sulfonated for 4 h and completely dried in a vacuum oven prior to imaging. Before sulfonation, the materials possessed a smooth surface, with only minor defects. Upon sulfonation, the surface of the hPB sample exhibited significant fragmentation, forming macroscopic cracks and visible surface distortion (Figure 7a). This distortion was associated with the inherent hydrophobic nature of hPB. As the reaction progressed, the introduction of hydrophilic sulfonic acid groups along the polymer backbone caused localized volume expansion and heterogeneous reaction kinetics, generating internal stress that led to surface cracking and deformation. These findings align with previous reports on sulfonation cross-linking of 3D-printed thick PE and PP parts,^36,37^ where diffusion-controlled reactions with sulfuric acid caused mismatched volumetric swelling along the diffusion pathway. For instance, in 15 mm PP gyroid samples with 15 wt % carbon fiber, surface cracks were observed after 2 h of sulfonation at 150 °C.^37^ The surface structure of the hPB-*g*-OH samples remained largely intact after sulfonation (Figure 7b–d), except for a macroscopic crack observed in the hPB-*g*-OH2 sample, likely caused during sample preparation for SEM imaging. This structural retention is attributed to the increased hydrophilicity of the hydroxyl-functionalized samples, which minimizes mismatched swelling between the pristine polyolefin and its sulfonated portions. This reduction in swelling mismatch decreases internal stress during the cross-linking process, preventing the surface fractures and/or cracks observed in the hPB sample. These findings suggest that cracking in polyolefin samples reported previously is primarily due to differences in hydrophilicity across different regions of the material. Controlling the hydrophilicity of polyolefins as carbon precursors can improve structural integrity while simultaneously enhancing sulfonation-induced cross-linking kinetics.

**Figure 7:**
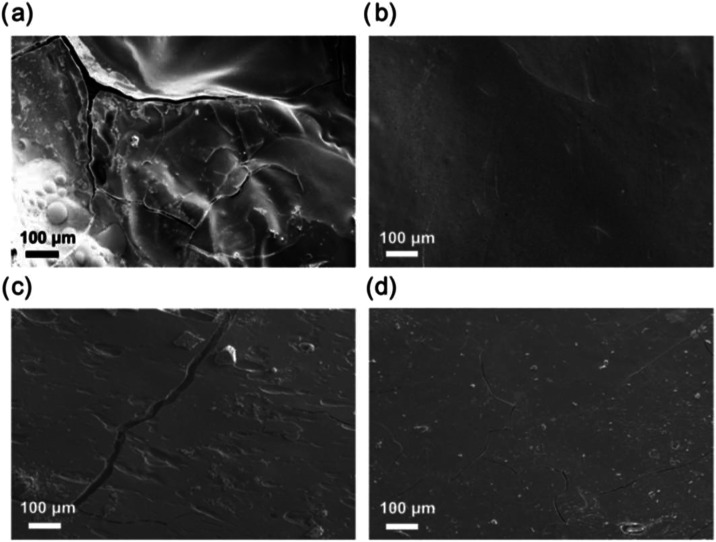
SEM micrographs after 4 h sulfonation times of (a) hPB, (b) hPB-*g-*OH1, (c) hPB-*g-*OH2, and (d) hPB-*g-*OH3.

The effect of sulfonation-induced crosslinking on carbon yield was also investigated across different samples, with sulfonation times varying from 15 min to 24 h. (Figure 8). For the hPB precursor, carbon yield values steadily increased with sulfonation time, obtaining a maximum carbon yield of 60 wt % after 24 h. The difference in carbon yield between functionalized and unfunctionalized hPB samples at lower sulfonation times can be attributed to the presence of hydroxyl groups, which alter their sulfonation cross-linking kinetics. These groups enhance hydrophilicity, facilitating faster cross-linking reactions and leading to improved carbon yield efficiency. The highest carbon yield values observed were 60 wt % for hPB, 58.6 wt % for hPB-*g-*OH1, 57.2 wt % for hPB-*g-*OH2, and 59.4 wt % for hPB-*g-*OH3. These results suggest that the incorporation of hydroxyl groups on the polymer backbone does not significantly impact the overall char yield, despite a reduction in the relative carbon atom content within the precursor. Furthermore, it is important to note that the reduction in crystallinity as a function of the sulfonation time for these samples (Figure 6) does not fully correlate with carbon yield measurements. This discrepancy suggests that crystallinity loss is primarily driven by the introduction of bulky functional groups, which disrupt chain packing and hinder crystallization, rather than being directly linked to cross-link formation.

**Figure 8:**
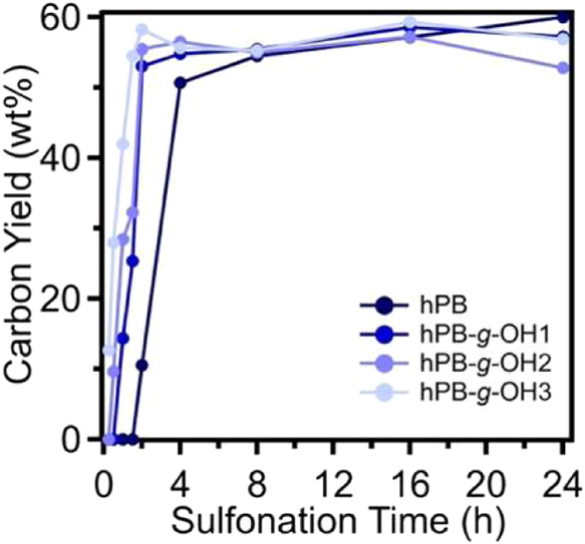
Carbon yield of hPB, hPB-*g-*OH1, hPB-*g-*OH2, and hPB-*g-*OH3 samples as a function of sulfonation time.

To further investigate the impact of precursor design on the porosity of hPB-derived carbon samples, nitrogen physisorption measurements were conducted at 77 K on samples that were sulfonated for 24 h and subsequently pyrolyzed. Nitrogen adsorption–desorption isotherms for these samples can be found in Figure 9a. All samples possessed type II isotherms with a H4 hysteresis loop indicative of the presence of micro-, meso-, and macropores,^50,51^ suggesting that all samples are porous in nature after carbonization. Pore size distributions were determined through nonlocal density functional theory (NLDFT), which is a method that employs fluid density functional theory to create adsorption isotherms in ideal pore structures.^52^ The pore size distribution results, shown in Figure S6, display that the carbon sample derived from hPB exhibited a narrow distribution peak centered around 1 nm, whereas the hPB-*g*-OH1, hPB-*g*-OH2, and hPB-*g-*OH3-derived carbon samples displayed a much broader distribution of micropore and mesopore sizes. This could be due to increased crosslinking efficiency of the hydroxyl functional materials, which would lead to reduced micropore formation upon carbonization as a result. The micropore volumes, determined through the t-plot method, were calculated to be 0.22 cm^3^/g for hPB, 0.20 cm^3^/g for hPB-*g-*OH1, 0.19 cm^3^/g for hPB-*g*-OH2, and 0.19 cm3/g for hPB-*g-*OH3 (Figure S7). This indicates that the micropore volume was not significantly affected by the presence of hydroxyl substituents, as it only slightly decreased with an increase in hydroxyl functionality. Moreover, the linear region of the BET plot was utilized to determine the monolayer capacity, which was then used to calculate the apparent surface area. It was observed that the BET surface area of the materials decreased with the addition of hydroxyl functionality, reducing from 702 m^2^/g for hPB to 607 m^2^/g for hPB-*g-*OH3 (Figure 9b). These results suggest that increasing hydroxyl functionality leads to a reduction in apparent surface area and micropore volume, likely due to enhanced cross-linking and structural densification, which limits micropore formation.

**Figure 9:**
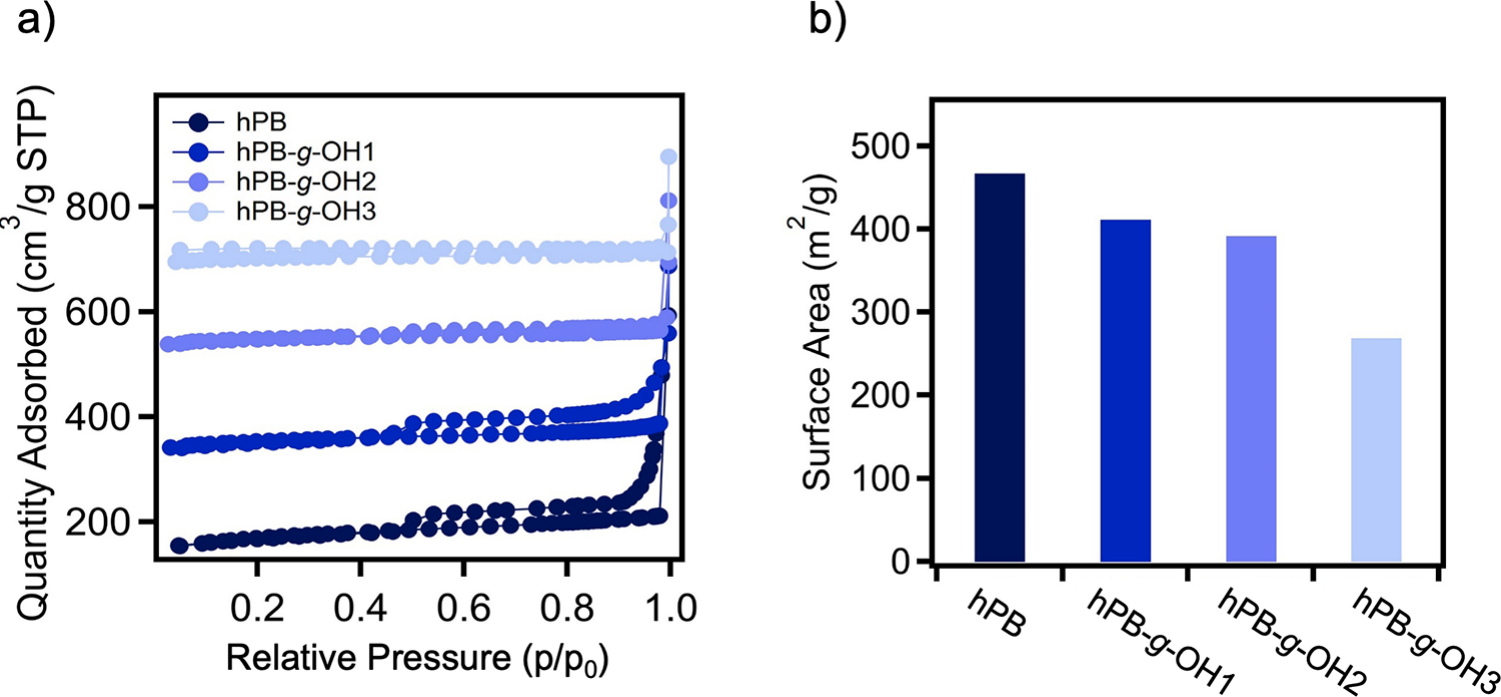
(a) Nitrogen physisorption isotherms of carbon samples from different hPB and hPB-*g-*OH precursors, vertically offset by 200, 400, and 600 cm^3^/g for hPB-*g*-OH1, hPB-*g*-OH2, and hPB-*g*-OH3, respectively. (b) Surface area values for different carbon samples derived from various precursors. All samples were sulfonated for 24 h.

## 4 Conclusions

The use of polyolefins as precursors for carbon materials synthesis presents a promising approach for cost-effective carbon production and plastic waste upcycling. However, challenges such as slow sulfonation-induced crosslinking kinetics could hinder their scalability for practical applications. This study demonstrates that hydroxyl functionalization of polyolefin backbones enhances sulfonation-induced crosslinking by improving hydrophilicity, thereby facilitating acid diffusion. As a result, this enables faster reaction kinetics and reduces microcrack formation. Moreover, it was found that the hydroxyl group incorporation on polymer backbones had a minimal impact on the overall char yield. We further reported the impact of polyolefin hydrophilicity on the pore texture of their derived carbon materials including surface area and pore size distribution. These findings highlight the potential of tailored polyolefin precursors for scalable and efficient carbon material production with an improved reaction efficiency and structural integrity.

## Supplementary Material



## Notes

^1^H NMR and WCA estimation of hydroxyl content; DSC thermograms of hPB, hPB-*g*-OH1, hPB-*g*-OH2, and hPB-*g*-OH3; second heat DSC thermograms of hPB materials after different sulfonation periods; FTIR spectra of materials after 24 h sulfonation-induced cross-linking; SEM micrographs of hPB, hPB-*g*-OH1, hPB-*g-*OH2, and hPB-*g*-OH3 before sulfonation-induced cross-linking; pore size distributions of hPB, hPB-*g*-OH1, hPB-*g-*OH2, and hPB-*g*-OH3 after 24 h sulfonation and subsequent carbonization; Micropore volumes of 24 h sulfonated hPB and hPB-*g*-OH materials after carbonization (PDF)
