# Supplementary material for: Tuning Polyolefin Hydrophilicity to Control Sulfonation Cross-Linking Kinetics for Carbon Synthesis
Source: ACS Appl Eng Mater. 2025 May 15;3(6):1736–44. doi: 10.1021/acsaenm.5c00210 (PMC12210215; doi:10.1021/acsaenm.5c00210)
Supplement: Supplementary file 1 [file em5c00210_si_001.pdf]

Supporting Information:

Tuning polyolefin hydrophilicity to control sulfonation crosslinking kinetics for carbon synthesis

Carmen B. Dunn[◊,1], Zoe Gunter[◊,1], Anthony Griffin[1], Paul Smith[1], Ahmed Al-Ostaz[2], Mine G. Ucak-Astarlioglu[3], Zhe Qiang[*,1]

[1]School of Polymer Science and Engineering, University of Southern Mississippi, 118 College Drive, Hattiesburg, Mississippi 39406, United States

[2]Department of Civil Engineering, University of Mississippi, Mississippi 38677, United States

[3]US Army Engineer Research and Development Center, 3909 Halls Ferry Road, Vicksburg, Mississippi 39180-6199, United States

*Corresponding Author: Z.Q. (zhe.qiang@usm.edu)

[◊]Co-first authors: C.B.D. and Z.G.

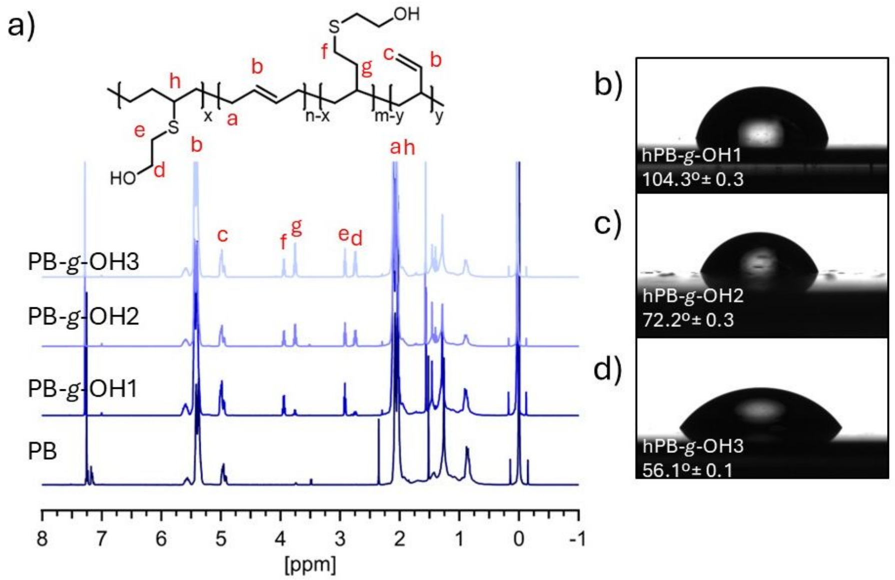

Figure S1. a) ^{1}H NMR spectra of lightly functionalized 32,000 g/mol PB, PB-*g*-OH1 (0.96 mol% -OH), PB-*g*-OH2 (4.63 mol% -OH), and PB-*g*-OH3 (5.63 mol% -OH) and water contact angle measurements for films of b) PB-*g*-OH1, c) PB-*g*-OH2, and d) PB-*g*-OH3.

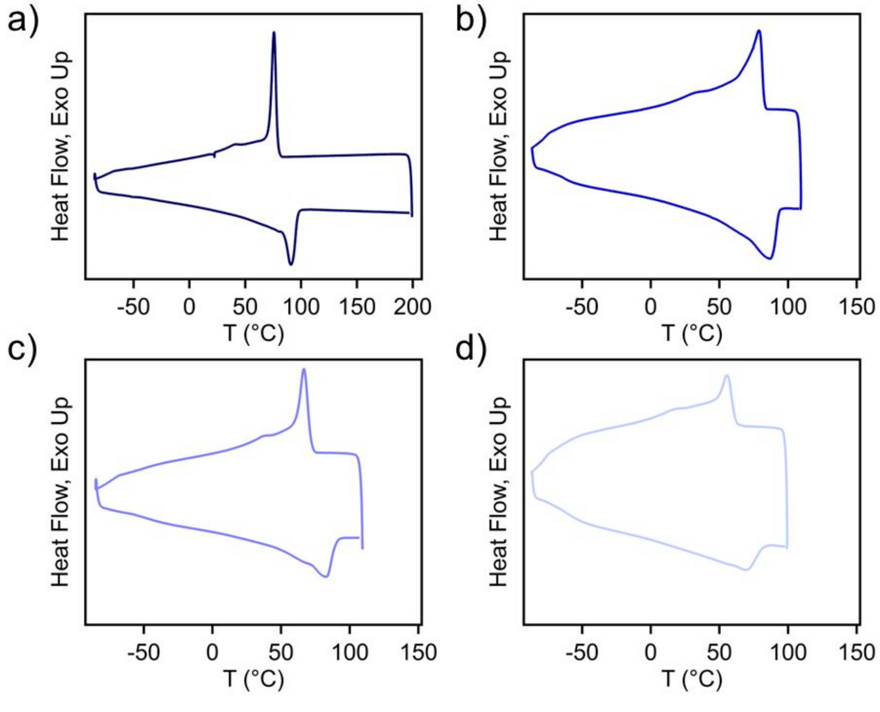

Figure S1. DSC thermograms of cooling (top) and heating (bottom) curves at 10 °C/min ramp rates for a) hPB, b) hPB-*g*-OH1, c) hPB-*g*-OH2, and d) hPB-*g*-OH3 demonstrating differences in crystallization behaviors.

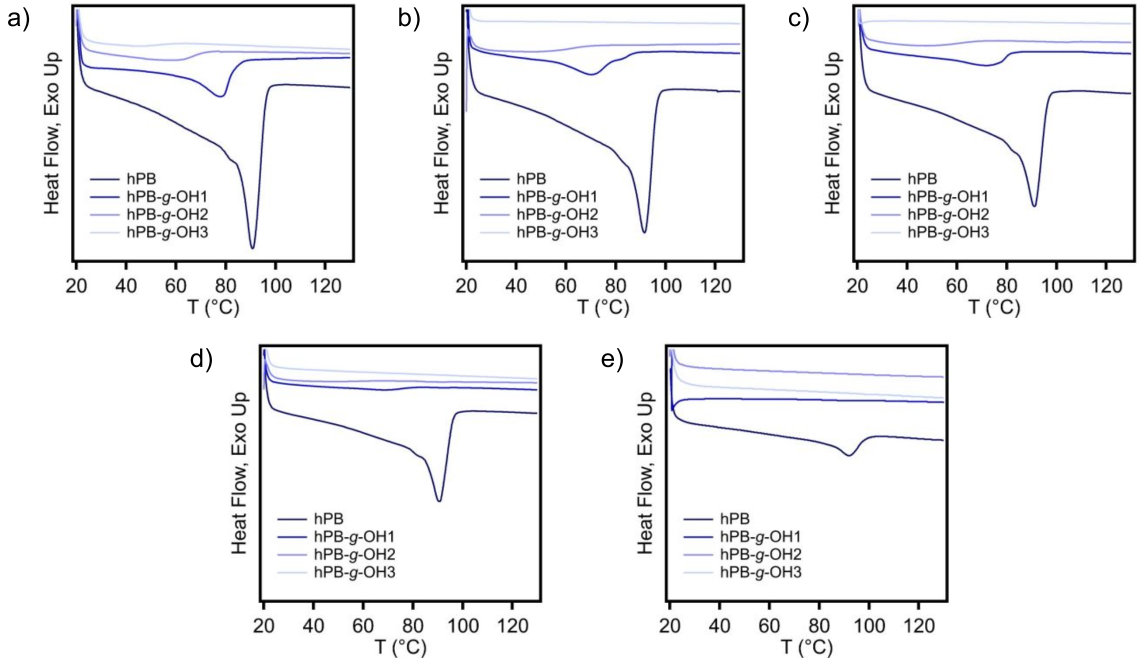

Figure S3. Second heat DSC thermograms of hPB materials after sulfonating for a) 15 min, b) 30 min, c) 1 h, d) 1.5 h, and e) 4 h.

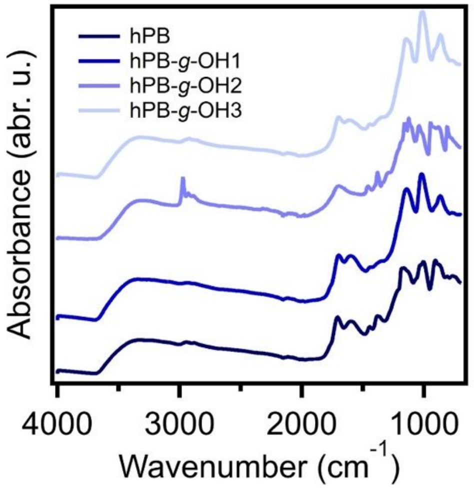

Figure S4. FTIR spectra of materials after 24 h of sulfonation-induced crosslinking

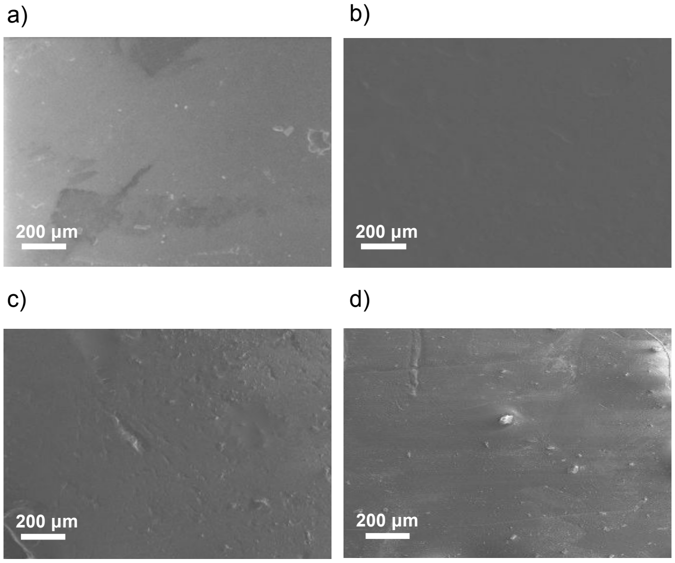

Figure S5. SEM micrographs of a) hPB, b) hPB-*g*-OH1, c) hPB-*g*-OH2, and d) hPB-*g*-OH3 before sulfonation induced crosslinking reaction.

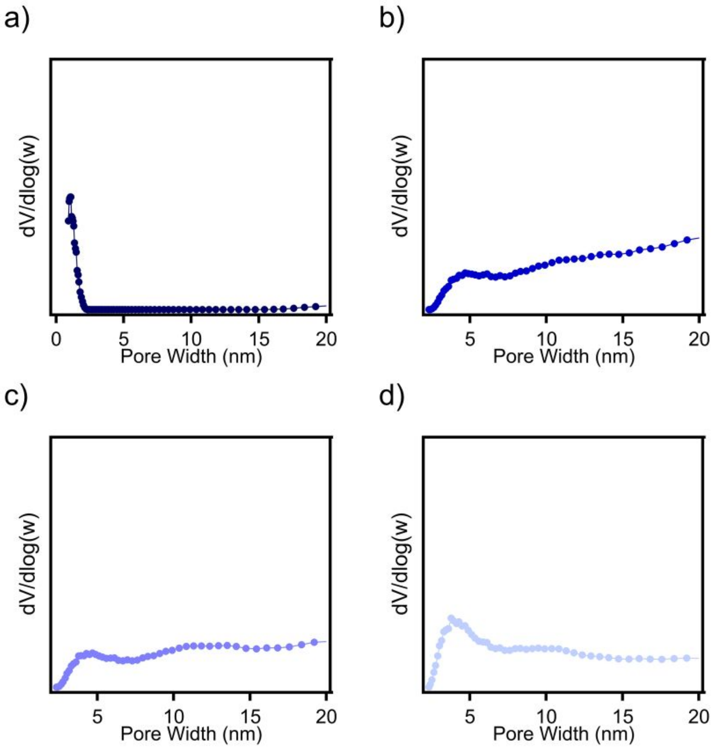

Figure S6. Pore size distributions of a) hPB, b) hPB-*g*-OH1, c) hPB-*g*-OH2, and d) hPB-*g*-OH3 after being sulfonated for 24 h and subsequently carbonized.

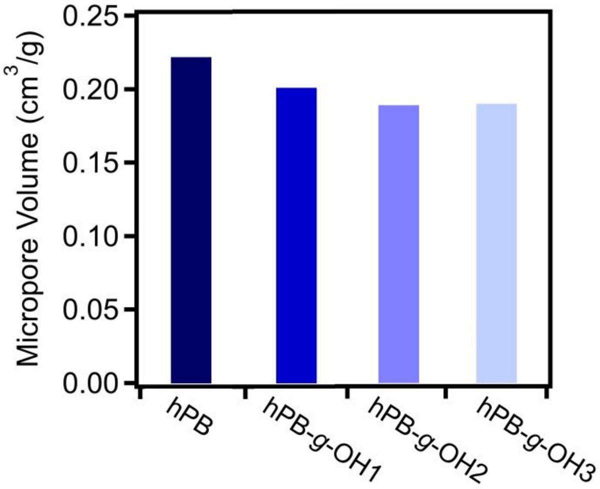

Figure S7. Micropore volumes of 24 h sulfonated hPB and hPB-OH materials after carbonization.
